# Effects of Different Processing Treatments on Almond (*Prunus dulcis*) Bioactive Compounds, Antioxidant Activities, Fatty Acids, and Sensorial Characteristics

**DOI:** 10.3390/plants9111627

**Published:** 2020-11-23

**Authors:** Ivo Oliveira, Anne S. Meyer, Sílvia Afonso, Alex Sequeira, Alice Vilela, Piebiep Goufo, Henrique Trindade, Berta Gonçalves

**Affiliations:** 1 Centre for the Research and Technology of Agro-Environmental and Biological Sciences—CITAB, Universidade de Trás-os-Montes e Alto Douro (UTAD), Quinta de Prados, 5000-801 Vila Real, Portugal; safonso@utad.pt (S.A.); pgoufo@utad.pt (P.G.); htrindad@utad.pt (H.T.); bertag@utad.pt (B.G.); 2 Department of Biotechnology and Biomedicine, Technical University of Denmark, DTU Building 221, DK-2800 Kgs, 2800 Lyngby, Denmark; asme@dtu.dk; 3 Universidade de Trás-os-Montes e Alto Douro (UTAD), Quinta de Prados, 5000-801 Vila Real, Portugal; alexasequeira94@gmail.com; 4 Biology and Environment Department, CQ-VR, Chemistry Research Centre–Vila Real, Food and Wine Sensory Lab, University of Trás-os-Montes and Alto Douro, 5001-801 Vila Real, Portugal; avimoura@utad.pt

**Keywords:** *Prunus dulcis*, processing, sensorial analysis, fatty acids, antioxidant

## Abstract

Almond is one of the most commonly consumed nuts worldwide, with health benefits associated with availability of bioactive compounds and fatty acids. Almond is often eaten raw or after some processing steps. However, the latter can positively or negatively influence chemical and sensorial attributes of almonds. This work was carried out to assess the effects of two processing treatments, namely; roasting and blanching on (i) contents of bioactive compounds, (ii) contents of fatty acids (3) antioxidant activities (4), sensorial characteristics of four neglected Portuguese almond cultivars (Casanova, Molar, Pegarinhos and Refêgo) and two foreign cultivars (Ferragnès and Glorieta). Results showed that in general, levels of bioactive compounds and antioxidant activities increased with roasting and decreased with blanching. Fatty acid profiles of raw kernels of all cultivars were generally identical although Refêgo exhibited a high content of α-linolenic acid. Following roasting and blanching, content of polyunsaturated fatty acids increased while saturated fatty acids, monounsaturated fatty acids and several health lipid indices decreased. Roasting positively affected perception of skin color and sweetness of Ferragnès and Glorieta as well as skin roughness of Molar and Pegarinhos. Blanching on the other hand led to positive changes in textural properties of Refêgo and Pegarinhos. This study reveals the nutritive benefits of consuming neglected almond cultivars in Portugal, and the novel data reported here could be of interest to growers, processing companies and consumers.

## 1. Introduction

The consumption of nuts, including almonds, is associated with some positive health benefits such as antioxidant capacities, anticancer and antiatherogenic actions, as well as the regulation of immune and inflammatory responses [1]. The health benefits of almond are related to the availability of unsaturated fatty acids [2] and polyphenols which are known to improve human health [3]. Although almonds are mainly eaten in the raw state, sliced, or roasted, almonds can also be processed to obtain products such as marzipan, butter, milk, and oil [4]. Besides their direct consumption, almonds are added to several sweet or savory dishes and food products for special purposes, such as improve complexion or texture. Two major methods used in the processing of almonds are roasting and blanching [5]. Roasting and blanching can significantly alter the physical, chemical, and nutritive properties of the almond kernel, thus resulting in desired changes in texture, color, flavor, aroma, and taste [6]. Positive changes are particularly evident in the case of the brittle roasted almond, whose pleasant color and aroma results from the decrease in moisture level observed after roasting [7]. Modifications at a microstructural level due to processing can also lead to unwanted changes such as lipid oxidation and nutrient loss [8]. Almond processing can also result in unpleasant odor, flavor, and color [9] which influences sensorial acceptance by consumers [10]. The chemical composition of almonds is greatly influenced by pre-harvest factors such as geographical location, physiological events, cultural practices, harvesting plans (e.g., the maturity stage of the fruit at harvest) and genotype often regarded as the most important [11,12]. Thus, it can be hypothesized that the effect of processing on the composition of almond will vary based on the cultivars. In the north of Portugal, there is a large area of almond cultivation which has been assigned a Protected Designation of Origin (PDO) status: Amêndoa Douro. The cultivars grown in Amêndoa Douro have been the subject of few scientific studies [13,14,15]. Preliminary data indicate that these cultivars are low-yielding due to unsuitable soils and environments as well as poor management practices [13]. Currently, new orchards are being planted with foreign almond cultivars, including the French cultivar Ferragnès and the Spanish cultivar Glorieta [16]. Lack of knowledge about the nutritive value of Portuguese cultivars has led to the assumption that these cultivars are of low quality. As a result, most growers and processing companies have lost interest in the Portuguese cultivars. The overall goal of this study was to determine and compare raw, roasted, and blanched Portuguese (cultivars Casanova, Molar, Pegarinhos, and Refêgo) and foreign cultivars (Ferragnès and Glorieta) concerning their nutritive value (fatty acid composition), eating qualities (sensorial characteristics) and bioactivities (content of bioactive compounds and antioxidant activities).

## 2. Results and Discussion

### 2.1. Content of Bioactive Compounds in Almond Cultivars

Significant cultivar differences were observed in the total phenolic and total flavonoid contents of raw kernels (Table 1). The total phenolic content ranged from 0.048 in Glorieta to 0.189 mg gallic acid equivalent (GAE)/g in Pegarinhos. The above range is similar to the range reported for other Portuguese cultivars (0.09–1.63 mg GAE/g; [17], but lower than the values reported for California (1.27–2.41 mg GAE/g; [18]) almonds. The values obtained for the total phenolic content in the present study are considerably different from the values obtained in our previous work using the same cultivars harvested in 2017 [14,15,16]. The above observation may be attributed to climate variability among the different growing seasons. Averagely, Portuguese cultivars had higher levels of phenolics (0.10 mg GAE/g) than the foreign cultivars (0.06 mg GAE/g in Ferragnès and 0.05 mg GAE/g in Glorieta). The total flavonoid content ranged from 0.35 in Pegarinhos to 1.86 mg catechin equivalents (CE)/g in Refêgo; the above values were considerably lower than those reported for the same cultivars harvested in previous years in Portugal [14]. Besides differences linked to the genotype, a strong influence of the harvest time is already known, suggesting that the synthesis of antioxidant compounds can occur in the last stage of ripening has already been reported. In the present work, all samples were harvested at commercial maturity, but slight variations on the ripening stage might be also linked to the differences between years.

### 2.2. Effects of Roasting and Blanching on the Content of Bioactive Compounds in Almond

Processing had a significant effect on the content of bioactive compounds in all almond cultivars (Table 1). Analysis of the results showed higher levels of phenolics in roasted kernels relative to raw kernels as observed in previous studies [19,20,21]. The above observation may be related to the potential increase in phenol extractability after the destruction of cellular structures in roasted kernels. In blanched almonds, the total phenolic content was generally reduced when compared to raw almonds although no significant effect was found for Casanova, Ferragnès, and Glorieta. Since blanched samples were also roasted, it may be assumed that the observed reductions in the total phenolic content in blanched kernels are due to skin removal. Indeed, 70–100% of almond phenolics is present in the skin [22] which is removed during blanching. Similarly, blanching led to reduced levels of flavonoids in the kernels. Accordingly, the total flavonoid content was similar for all cultivars after blanching but lower than contents in both raw and roasted samples. In general, roasting had no effect on the total flavonoid content of almond cultivars except for a 58% decrease for Molar and a 72% decrease for Refêgo. The above results are in contradiction to the increase in total phenolic content observed in all cultivars after roasting and might be an indication of the degradation of more complex phenolic structures such as polymerized proanthocyanidins and glycosylated flavonoids at high temperatures [19,20,21].

### 2.3. Antioxidant Activities of Almond Raw Extracts

The antioxidant potential of the six almond cultivars was evaluated using three different methods (ABTS, DPPH and β-carotene) which enabled elucidation of the mechanism of action. Raw extract data presented in Table 1 show that highest ABTS and DPPH activities were obtained with Pegarinhos, followed by Casanova. The highest percentage inhibition of lipid peroxidation (β-carotene-linoleic acid bleaching assay) on the other hand was obtained with Glorieta, Casanova and Ferragnès. Overall, the lowest antioxidant activities were recorded in Refêgo.

No correlations were found between the total flavonoid content and antioxidant activities. Nevertheless, positive correlations were found between the total phenolic content and ABTS (R^2^ = 0.7057, y = 50.67x + 1.7828) and between the total phenolic content and DPPH (R^2^ = 0.7892, y = 0.0298x – 0.0093). Such correlations have already been reported in previous works using the same almond cultivars [14,15,16] (and in studies using walnut [23].

### 2.4. Effects of Roasting and Blanching on the Antioxidant Activities of Almond Extracts

Even if not significant, the average DPPH and ABTS activities of all the extracts increased after roasting except for the DPPH for Refêgo which was reduced by 67%. As shown in Table 1, Ferragnès had the highest antioxidant activities after roasting followed by Casanova and Pegarinhos. Refêgo on the other hand exhibited the lowest activities. Additionally, Refêgo exhibited the lowest antioxidant activities after blanching. Blanching in general led to huge drops in DPPH and ABTS activities for all almond extracts. Indeed, the highest mean DPPH activity of 0.70 µg Trolox/g observed for Glorieta (statistically similar to Casanova, Molar, and Pegarinhos) blanched kernels was two times lower than that of the raw kernels; the highest mean ABTS activity of 0.68 µg Trolox/g observed for Pegarinhos (statistically similar to Ferragnès and Molar) blanched kernels was 17 times lower than that of the raw kernels. Results of the β-carotene-linoleic acid assay showed that roasting reduced the potential of almond samples to inhibit lipid peroxidation. The ability for almond to inhibit peroxidation was further reduced after blanching (Table 1).

Correlation analyses using data from roasted samples showed that ABTS (R^2^ = 0.708, y = −3.76362x + 14.27525) and DPPH (R^2^ = 0.545, y = −2.48231x + 16.18607) negatively correlated with the total phenolic content, a finding inconsistent with what was observed for raw samples. There were no significant correlations found with data from blanched samples. Negative correlations between phenolic levels and antioxidant activities were reported in few studies [24,25] linked these negative correlations to the unspecific nature of the Folin–Ciocalteu assay since the reagent targets all compounds with phenolic units with no consideration for the number of hydroxyl groups in a compound [26] or interfering compounds [27]. In the present study, negative correlations were likely due to different magnitudes of change due to roasting. For example, the 11000% increase in the total phenolic content of Refêgo after roasting was accompanied by only a 69% increase in the ABTS activity.

### 2.5. Fatty Acid Composition of Almond Cultivars

A total of 23 fatty acids were identified in the six almond cultivars. This number is similar to what was previously reported by Oliveira et al. (2019) [15] and Beyhan et al. (2011) [11]. Lower number of fatty acids in almonds have been reported in several studies [28,29]. These differences can be attributed mainly to genotype [30]. In the present study, 13 fatty acids exhibited high abundance and/or significant changes due to processing as shown in Table 2. In raw kernels, the most abundant fatty acid was C18:1 (elaidic + oleic acids), followed by C18:2 (linoleic + linolelaidic acids) except for Refêgo which had α-linolenic acid as the second abundant fatty acid. Other major fatty acids were α-linolenic and palmitic acids (Table 2). Most studies have reported oleic, linoleic, palmitic, and stearic acids [15,28,31,32,33,34] as the most abundant fatty acids in almond. The content of α-linolenic acid in almond is usually reported to be around 1%. Thus, the 16.21% of α-linolenic acid found in the present study for Refêgo can be considered high even when compared to the 11.00 % and 9.45% reported by Askin et al. (2007) [35] and Oliveira et al. (2019) [15], respectively. This finding is extremely relevant given the important role that α-linolenic acid plays in the reduction of diabetes and coronary heart diseases [36]. α-linolenic acid is also the precursor of long-chain polyunsaturated fatty acids in the human body (e.g., eicosapentaenoic and docosahexaenoic acids) which have positive impacts on prothrombotic risk factors [37]. However, oils rich in linoleic and linolenic acids have reduced oxidative stability and hence are more prone to rancidity; this consequently results in the production of undesirable volatile compounds and off-flavors [38]. Oils rich in oleic acid on the other hand have better resistance to oxidation, longer shelf life and higher nutritional values [39]. Differences in the fatty acid composition of the six almond cultivars can be related to several factors such as the genotype and growing temperature which are the most important [11,40].

### 2.6. Effects of Roasting and Blanching on the Fatty Acid Composition of Almonds

Although not always the case, both processing treatments (roasting and blanching) had similar effects on the fatty acid profile of almonds; however, the effect of blanching was stronger than that of roasting. After roasting, the major fatty acid in all cultivars remained C18:1 (elaidic + oleic acids). However, the second most abundant fatty acid in raw kernels (C18:2) was substituted in roasted kernels with α-linolenic acid. Indeed, there was a sharp increase in the level of α-linolenic acid in all cultivars after roasting except for Refêgo. Other major fatty acids identified in roasted kernels were erucic acid (third most abundant fatty acid in Casanova, Glorieta, and Pegarinhos), nervonic acid (third most abundant fatty acid in Ferragnès) and palmitic acid (second most abundant fatty acid in Refêgo and third in Molar). C18:1 (elaidic + oleic acids) and α-linolenic acid were also the most abundant fatty acids after blanching except for Molar which had erucic acid as the second most abundant compound. Other fatty acids found in high contents after blanching were palmitic acid (third major fatty acid in Casanova), erucic acid (second major fatty acid in Molar and third in Ferragnès, Pegarinhos, and Refêgo) and nervonic acid (third major fatty acid in Molar).

Both roasting and blanching led to decreases in the contents of C18:1 (elaidic + oleic acids) and C18:2 (linoleic + linolelaidic acids) with the exception of Refêgo where an increase in the content of C18:1 was found after roasting. In fact, C18:2 (linoleic + linolelaidic acids) was undetectable in Ferragnès after roasting. Several studies [6,37,41,42] have identified both increases and decreases in C18:1 levels after subjecting almonds to different processing treatments, although the changes observed in these studies were much lower in magnitude than those found in the present study. The content of palmitic acid generally decreased in almond samples after roasting and blanching (except for an increase for Molar and Refêgo after roasting and no change for Casanova, Glorieta and Molar after blanching). Research shows that roasting lead to increased levels of palmitic acid in almond [6,43]; the effect however, depends on the roasting temperature and time. In fact, the content of palmitic acid can be gradually reduced with prolonged exposure of kernels to high temperatures [41]. The content of stearic acid in almonds tended to decrease after roasting (except for an increase for Pegarinhos and Refêgo) and to increase after blanching (except for a decrease for Casanova and Refêgo).

Following roasting or blanching, the content of α-linolenic acid considerably increased in all cultivars, except for Refêgo, which showed an opposite response. Few studies found significant changes in the content of α-linolenic acid after processing of almonds: Ghazzawi and Al-Ismail (2017) [37] recorded a slight increase in the content of linolenic acid after frying, but not after roasting; Schlörmann et al. (2015) [42] did not detect this fatty acid in raw and roasted almond samples. In fact, the low level of linolenic acid in several cultivars is likely responsible for the lack of data regarding the effects of processing on almond lipids. In walnut, increases or decreases in the content of α-linolenic were found, depending on the roasting conditions [42,44]; In cashew, pistachio and pine nuts, these changes depended on the processing method [37]. In all cultivars, the amount of γ-linolenic acid rose or tended to increase after roasting (except for Refêgo in which it was not detected) and blanching (except for a decrease in Casanova). Additional fatty acids whose contents rose in all cultivars after roasting or blanching were erucic and nervonic acids. In the case of nervonic acid however, two exceptions were found: for a decrease in Refêgo after roasting, and a decrease in Ferragnès after blanching.

Interestingly, three long-chain polyunsaturated fatty acids are reported in this study that were undetected in raw almond kernels, and only emerge after processing. These included after roasting cis-11,14-eicosadienoic acid in. Casanova, Glorieta and Pegarinhos, cis-8,11,14-eicosatrienoic in Molar and Pegarinhos, andcis-5,8,11,14,17-eicosapentaenoic acid in Molar and Pegarinhos. With the exception of cis-11,14-eicosadienoic acid in Casanova, the three long-chain polyunsaturated fatty acids were detected in all cultivars following blanching.

### 2.7. Health Lipid Indices of Almond Cultivars

The content of saturated fatty acids (SFA) ranged from 4.36 % in Glorieta to 18.04 % in Molar (Table 3). SFA values were close to the threshold of 10 % [40] and similar to those reported for the same cultivars by Oliveira et al. (2019) [15]. Research shows that high-fat fruit rich in SFA are less susceptible to lipid oxidation and rapid deterioration than low-fat fruit. Similarly, Molar’s ability to resist oxidation than the other cultivars can be deduced from its high SFA content. Significant differences in cultivars relative to the content of monounsaturated fatty acids (MUFA) were also observed and values ranged from 65.58 % to 78.09 %, in agreement with previous studies [15,45]. The content of polyunsaturated fatty acids (PUFA) ranged from 16.37 % in Molar to 23.81 % (Table 3). It is reported that nuts with high levels of MUFA (oleic acid in particular) are more stable and less susceptible to oxidative rancidity than those with high levels of PUFA [29,39]. In the present study, PUFA/MUFA values were similar for all cultivars and lower than 1, indicating good oil stability. The UFA/SFA ratio is another parameter related to the shelf life of food products; the lower the UFA/SFA ratio, the higher the prospective shelf life of almonds [46]. In this study, the lowest UFA/SFA value of 4.57 % was calculated for Molar, which indicates its ability to withstand long storage periods. It is important to note that despite their relevance to the oxidative stability of almonds, SFA at high levels are harmful to the cardiovascular system [29].

The atherogenicity index (AI) is defined as the relationship between the main saturated (pro-atherogenic) and unsaturated (anti-atherogenic) fatty acids. The lower the AI values, the less likely the cardiovascular risk [47]. AI values were similar for all cultivars with the exception of Molar which obtained the highest value due to the abundance of SFA (Table 3). The thrombogenicity index (TI) indicates the propensity of lipids to form clots in blood vessels and is defined as the relationship between saturated (pro-thrombogenetic) and unsaturated (MUFAs, PUFAs—n6, and PUFAs—n3; anti-thrombogenetic) fatty acids. The lower the TI values, the healthier the oil or the fat contained in a food (Ulbricht and Southgate, 1991). The “cultivar” factor significantly affected TI and the highest value of 0.16 was calculated for Molar. The hypocholesterolemic/hypercholesterolemic (h/H) ratio estimates the functional role of fatty acids in the metabolism of lipoproteins involved in the transport of plasmatic cholesterol. Thus, the h/H can be used as an indicator for the risk level of cardiovascular disease incidence [48] (Santos-Silva et al., 2002). Glorieta obtained the highest h/H value indicating its ability to contribute to improved cardiovascular health.

### 2.8. Effects of Roasting and Blanching on Almond Health Lipid Indices

With some exceptions, blanching similarly affected health lipid indices of all almond cultivars (Table 3). Casanova, Ferragnès, Glorieta, Molar, and Pegarinhos exhibited similar responses after roasting, in contrast to Refêgo. Besides Casanova and Glorieta in which increases were observed, there was a decrease in the content of SFA in almond cultivars after roasting. Furthermore, almond cultivars showed a decreasing trend in SFA content after blanching with the exception of Glorieta and Refêgo. The content of MUFA generally decreased in almond samples after roasting and blanching except for Refêgo after roasting; the inverse was true for the PUFA content. The most common responses observed after nut processing are increases in SFA [37,41,42] and decreases in PUFA and MUFA [37] levels. In the study by Valdés et al. (2015) [6], the contents of SFA and MUFA increased while that of PUFA decreased in almond after processing. In the present study, both increases and decreases were found in relation to the contents of SFA, MUFA, and PUFA. Beside the “cultivar” effect, these variations are most likely related to processing conditions and basal levels of all fatty acids, but also to the fact that minor fatty acids are not usually quantified by investigators.

Similar to the PUFA content, an increasing trend was observed in relation to the PUFA/MUFA ratio of all cultivar after processing with the exception of that of Refêgo after roasting. The lowest PUFA/MUFA value obtained after roasting was found for Refêgo. In the case of blanching, both Refêgo and Casanova achieved the lowest PUFA/MUFA. The above observation indicates that these cultivars are most likely to be less prone to oxidation after processing. After roasting, the UFA/SFA ratio increased in four cultivars (Ferragnès, Molar, Pegarinhos, and Refêgo) and decreased in two cultivars (Casanova and Glorieta). Both Casanova and Refêgo attained the lowest UFA/SFA values, as also observed with the PUFA/MUFA values. Blanching differentially affected the UFA/SFA ratio: The UFA/SFA ratio calculated for Refêgo and Glorieta decreased whereas an increase was recorded for Ferragnès and Molar. Casanova and Pegarinhos on the other hand showed no significant changes relative to the UFA/SFA ratio.

Health lipid indices were significantly affected by both processing treatments. The AI and TI values decreased or tended to decrease in all cultivars with the exception of Refêgo in which an increase was observed after roasting. The h/H ratio on the other hand generally increased after roasting and blanching although some exceptions were observed. For instance, a decrease in the h/H ratio was observed for Refêgo and Glorieta after roasting and blanching respectively. In roasted kernels, AI and TI values were similar for all cultivars with the exception of Refêgo which obtained high values. These high values of AI and TI observed for Refêgo can be associated with increased risks to cardiovascular problems; indeed, Refêgo exhibited the lowest h/H value after roasting. Overall, the highest h/H value was calculated for the oil extracted from roasted kernels of Pegarinhos.

### 2.9. Sensorial Analysis of Raw Almond Samples

**Figure 1:**
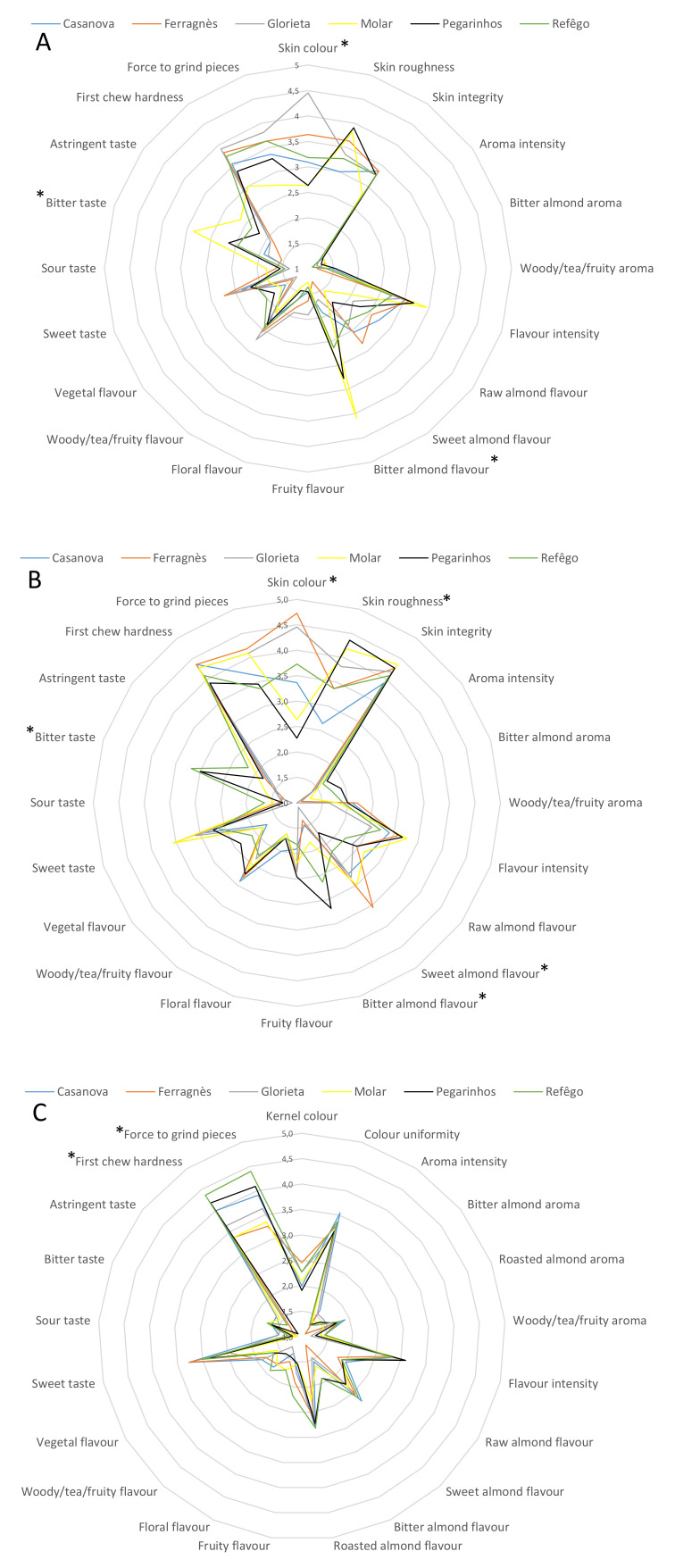
Spider plot of the sensory profile of raw (**A**), roasted (**B**) and blanched (**C**) almond kernles. Asterisks (*) indicate represent significant differences among cultivars *p*  <  0.05, ANOVA Tukey’s test.

The sensory profiles of raw kernels from all almond cultivars studied were similar to that shown in Figure 1A. Significant differences were observed relative to skin color, bitter almond flavor and bitter taste. Skin color was found to be darker in the foreign cultivars (Glorieta and Ferragnès) than the Portuguese cultivars. Bitter almond flavor and bitter taste were more associated with Molar and Pegarinhos than the rest of the cultivars. The primary taste characteristics easily identified by consumers are sweetness and astringency followed by bitterness or sourness [49]. The bitter taste derived from eating raw kernels of Molar and Pegarinhos might be due to a high concentration of benzaldehyde in these cultivars. Indeed, benzaldehyde is a chemical with a bitter taste and a low odor threshold [50]. Several Portuguese almond cultivars including Pegarinhos have high levels of benzaldehyde [14].

### 2.10. Effects of Roasting and Blanching on Sensory Characteristics of Almonds

The main sensory attributes differentiating the six almond cultivars after roasting were found to be skin color, bitter almond flavor, bitter taste, skin roughness, and sweet almond flavor (Figure 1B). Skin color rated highest for the foreign cultivars (Ferragnès and Glorieta) and lowest for Molar and Pegarinhos. Relative to skin roughness, the skin of Molar and Pegarinhos were found to be rough whereas that of Casanova was found to be smooth. The sweet almond flavor was strongly associated with Ferragnès and barely perceived for Pegarinhos. Pegarinhos and Refêgo achieved the highest rating for bitter almond flavor and bitter taste whereas Glorieta was rated lowest for bitter almond flavor. Roasting is known to increase the levels of compounds such as pyrazines, furans, and pyrrols in food products. The above compound represents three groups of chemicals with nutty and roasted aromas [50,51] that are formed through non-enzymatic Maillard browning reactions [52]; these compounds are also known to enhance the aromas of roasted almonds [40]. In our previous work [14], pyrazines were not detected in any of the six cultivars studied; it was, however, detected in the Portuguese cultivar Amendoão. Xiao et al. (2014) [53] reported decreases in the levels of benzaldehyde and several alcohols in food products after roasting. For the six cultivars object of the present study an increase in the amount of benzaldehyde was observed after roasting. The above observation may partly account for the significant differences among cultivars relative to bitter sensorial characteristics.

In the case of blanched kernels, differences in sensorial characteristics among cultivars were only recorded for textural parameters, namely first chew hardness and force to grind pieces (Figure 1C). Both characteristics rated highest for Refêgo and Pegarinhos and lowest for Ferragnès and Molar. These changes in textural parameters are likely due to the low water content of blanched kernels which were subjected to two processing steps both involving heating. Indeed, water is known to directly influence the hardness, crispness, crunchiness and toothpack attributes of almonds [54]. Consumers usually show a preference for crispy, crunchy [54,55], and sweet almonds [55]. Hence, good ratings by the panelist for blanched kernels relative to textural properties could have positive implications for the almond industry. Indeed, hard, fracturable and crunchy almonds are used as cooking ingredients to improve food texture while moist and chewy almonds are suitable for beverages [56].

### 2.11. Multivariate Analyses of Bioactive Compounds, Antioxidant Activities and Fatty Acids Data

**Figure 2:**
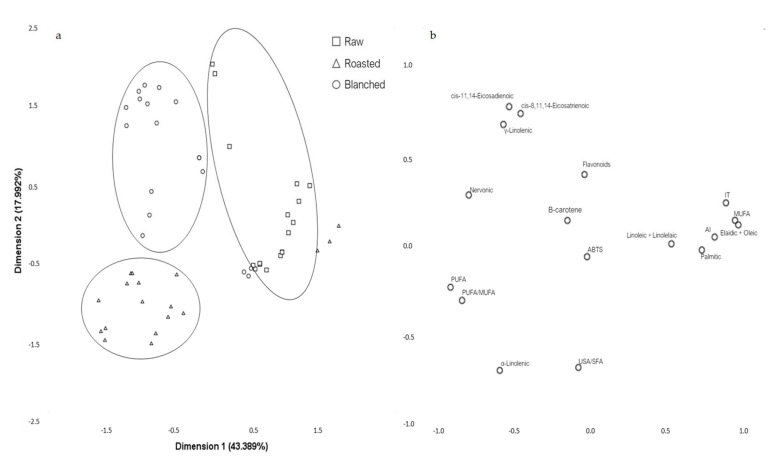
Principal component analysis of bioactive compounds, antioxidant activities and fatty acids data from raw, roasted and blanched almond kernels: scores plot of the first and second principal components (**a**) showing the clustering of cultivars and treatments; loadings plot (**b**) reflecting the influence of parameters on the separation of samples.

All data obtained (with the exception of those from sensorial analyses) were submitted to linear discriminant analysis (LDA) to identify parameters that could be used to differentiate both the almond cultivars and the processing methods. Seventeen variables which differed significantly between cultivars and processing treatments were selected using LDA and subjected to Principal component analysis (PCA) (Figure 2). PCA generated a 17-component model that explained 100% of the total variance in the data. The first and second components accounted for 43.398% and 17.992% (for a total of 61.318%) of the total variance respectively (Figure 2a). All cultivars and processing methods were well separated on the PCA except for roasted and blanched kernels of Refêgo and Casanova respectively. Indeed, the loading plot in Figure 2B showed that palmitic acid, MUFA, AI and TI contributed the most to the separation of Refêgo from other cultivars. Blanched kernels of Casanova on the other hand contained high levels of C18:1 (elaidic + oleic acid), palmitic acid, MUFA and AI and low levels of α-linolenic acid.

## 3. Materials and Methods

### 3.1. Almond Samples and Processing Treatments

Almond samples were obtained in 2019 from growers in the municipality of Torre de Moncorvo (Northeastern Portugal, 41°10′26″ N 7°3′0″ W), and were constituted of fruit of the Portuguese traditional cultivars (Casanova, Molar, Pegarinhos and Refêgo), French cultivar Ferragnès and Spanish cultivar Glorieta. Samples from each cultivar were harvested at commercial maturity and subdivided into three parts to obtain three replicates. Before processing, almond fruit were deshelled to obtain raw kernels which included the skin. Medium roasting of the kernels was conducted at 138 °C for 33 min [53]. Blanching was performed using the method described by Milbury et al. [18] with some modifications; kernels were immersed in boiling water for 30 s and the skins were removed by hand. Deskinned kernels were left to dry at room temperature and this was followed by roasting at 138 °C for 33 min. These treatments were selected due to their extensive use in industrial processing of almonds. Raw, roasted, and blanched kernels were finely ground to obtain a fresh flour for chemical analyses.

### 3.2. Bioactive Compounds: Total Phenolic and Total Flavonoid Contents

An “antioxidant extract” was prepared by vortex-mixing 40 mg of the sample with 1 mL of 70 % methanol. The mixture obtained was heated for 30 min at 70 °C, and then centrifuged (Eppendorf Centrifuge 5804 R, Hamburg, Germany) at 25,200 rcf for 15 min at 1 °C. The supernatant which constituted the “antioxidant extract” was filtered (Spartan filters 0.2 mm) into HPLC amber vials and used for the determination of the total phenolic content, total flavonoid content, and antioxidant activities. A slight modification of the methodology of Singleton and Rossi [26] (1965) was used for quantification of total phenolics as follows: 20 µL of the “antioxidant extract” was mixed with 100 µL of Folin-Ciocalteu phenol reagent (1:10 v/v/in bidistilled H2O) and 80 µL of 7.5% Na2CO3 in a 96-well microplate (Multiskan™ FC Microplate Photometer, Waltham, MA, USA). The microplate was incubated in the dark for 15 min at 45 °C. Afterward, the absorbance values against the blank value were recorded at 765 nm in a microplate reader (Multiskan GO Microplate Spectrophotometer, Thermo Scientific, Vantaa, Finland). Simultaneously, gallic acid solutions of different concentrations were analyzed and a calibration curve was constructed for quantitation purposes. The total phenolic content was expressed as mg gallic acid equivalent (GAE)/g f.w (fresh weight). The total flavonoid content was determined using the colorimetric method described in Dewanto et al. [21] with some modifications. In a 96-well microplate, 25 µL of “antioxidant extract” was combined with 100 µL of water and 10 µL of NaNO2. The microplate was placed in the dark at room temperature. After 5 min, 15 µL of 10% AlCl3 was added to the wells and the microplate was incubated again at room temperature in the dark for 6 min. Then, 50 µL of NaOH 1M and 50 µL of water were added. The absorbance values against the blank value were recorded at 510 nm. Simultaneously, catechin solutions of different concentrations were analyzed. The total flavonoid content was quantified using the calibration curve of catechin and expressed as mg catechin equivalent (CE)/g f.w.

### 3.3. Antioxidant Activities

The 2,2′-azino-bis(3-ethylbenzothiazoline-6-sulfonic) acid (ABTS) radical scavenging activity was evaluated in a 96-well microplate using the method of Re et al. [57]. An ABTS radical solution was prepared by mixing 7 mM of ABTS at pH 7.4 (5 mM NaH2PO4, 5 mM Na2HPO4, and 154 mM NaCl) with 2.5 mM K2S2O8. After incubation in the dark at room temperature for 16 h, the ABTS solution was diluted with ethanol until an absorbance of 0.70 ± 0.02 units at 734 nm obtained. In each microplate well, 15 µL of the “antioxidant extract” was combined with 285 µL of freshly prepared ABTS solution. The mixtures were incubated at room temperature in the dark for 10 min and absorbance values were measured at 734 nm. ABTS activity was expressed using the linear calibration curve of trolox as g trolox equivalent/g f.w. The 2,2-diphenyl-1-picrylhydrazyl (DPPH) antioxidant activity assay was performed by spectrophotometry as described by Siddhraju & Becker [58]. Briefly, 20 μL of the “antioxidant extract” and 280 μL of freshly prepared methanolic radical DPPH solution (6 × 10–5 mol/L) were introduced into a 96-well microplate. The microplates were covered with a foil paper and left for 30 min at room temperature. The reduction in absorbance was measured at 517 nm. The DPPH activity was expressed using the linear calibration curve of trolox as g trolox equivalent/g f.w. To assess the ability of almond extracts to inhibit lipid oxidation, the β-carotene-linoleic acid bleaching assay was performed using the method described by Salleh et al. [59] with minor modifications as follows: a mixture of β-carotene and linoleic acid was prepared by adding 0.5 mg β-carotene with 1 mL chloroform (HPLC grade), 25 µL linoleic acid and 200 mg Tween 40. After complete evaporation of the chloroform under vacuum, 100 mL of water was added to the residue which was gently stirred to form a yellowish emulsion. To 50 µL of the “antioxidant abstract” were added 0.25 µL of the yellowish emulsion. After thorough mixing, the mixture was incubated in a water bath at 50 °C for 2 h followed by the measurement of absorbance values at 470 nm against a blank. Percentage inhibition (I%) of lipid peroxidation was calculated using the following equation:

I% = (Aβ-carotene after 2 h/Ainitial β-carotene) × 100;(1)

where Aβ-carotene after 2 h is the absorbance value of β-carotene after 2 h of incubation and Ainitial β-carotene is the absorbance value of β-carotene before incubation.

### 3.4. Fatty Acids

Total oil was extracted from 5 g almond sample with petroleum ether in a Soxhlet apparatus [30] operating at 135 °C for 2 h. The extracted oil was used to prepare methyl esters of the corresponding fatty acids (FAME) according to EEC (1991) [60]. The resulting FAMEs were analyzed with a Shimadzu GC-2010 Plus gas chromatograph (Shimadzu, Kyoto, Japan) equipped with a flame ionization detector (FID-2010 Plus). Component peak separation was obtained on a DB-225MS capillary column (0.25 μm, 30 m × 0.25 mm i.d., Agilent Technologies, Wilmington, DE, USA). Helium was used as the carrier gas (200 kPa, constant flow, 1 mL/min). The temperature of the column was maintained for 10 min at 200 °C and then increased to 220 °C at 5°C per min. The inlet and detector temperatures were set at 270 °C. The split ratio was 5:1 and the injected volume was 1.0 µL. The constituent FAMEs were identified by comparison with standard FAME mixtures (FAME 37, Supelco, Bellefonte, PA, USA). The amount of each FAME was expressed as a weight percentage (%) of the total FAMEs represented in the chromatogram, with the assumption that no other major lipids and other substances were present in the almond oils. In several samples, the peaks for elaidic and oleic acids co-eluted, as well as the peaks for linoleic and linolelaidic acids. Thus, peak areas corresponding to these compounds were summed in the calculations. Fatty acid data was used for the calculation of several health lipid indices. The atherogenic index (AI) and the thrombogenic index (TI) were calculated using the equations proposed by Ulbricht and Southgate [47].

The hypocholesterolemic/hypercholesterolemic (h/H) index indicates the sample’s potential to provide good over bad cholesterol and was calculated using the equation proposed by Santos-Silva et al. [48].

### 3.5. Sensorial Analysis

Raw, roasted, and blanched almond kernels were evaluated by a panel of 12 tasters from Departamento de Biologia e Ambiente (DeBA-ECVA), Universidade de Trás-os-Montes e Alto Douro in Portugal. The panelist was well trained and have participated in sensory tests from previous studies. The test took place between 4:00 pm and 5:00 pm in a room with regulated temperature and air pressure/flow. Almond kernels were presented to the tasters in white pyrex dishes. The testing environment and procedure/equipment were all following ISO 8589:2007. Three tasting sessions were carried out; the first, second and third sessions were done using raw, roasted, and blanched almonds respectively. A Quantitative Descriptive Analysis (QDA) was performed using 20 descriptors adapted from Civille et al. [49] and a structured scale from 1 (least intense) to 5 (most intense) for each descriptor (ISO 4121:2003).

### 3.6. Statistical Analyses

Data are presented as mean (f.w) of three replicates. Differences among means were determined by analysis of variance (ANOVA) using SPSS (Statistical Package for Social Sciences) 19.0 (IBM Corporation, New York, NY, USA). The fulfillment of ANOVA requirements, namely the normal distribution of the residuals and the homogeneity of variance was evaluated using the Shapiro–Wilk’s test (n < 50) and the Levene’s test, respectively. The Tukey test was used for the comparison of means which were considered different at a 5% significance level. A stepwise linear discriminant analysis (LDA) was performed to find the linear combination of parameters that best characterized raw, roasted, or blanched samples. The LDA involved the use of a combination of forward selection and backward elimination procedures for variable separation. Before selecting a new variable to be included in the model, it was ascertained that all previously selected variables were still significant which enabled identification of all possible significant variables. The Wilk’s lambda test was applied for variable selection through verification of the significance of each canonical discriminant functions using the probabilities F = 3.84 to add and F = 2.71 to remove. To avoid overoptimistic data modulation, the model performance was assessed using a leave-one-out cross validation procedure. Principal component analysis (PCA) was performed by plotting all data in a multidimensional space using LDA significant variables as dimensions (factor scores). With this approach, the number of variables was reduced to a smaller number of newly derived variables (principal component or factors) that adequately summarized the original information, and highlighted underlying patterns in the data collected. Scree plots were used for retaining the most useful factors taking into consideration eigenvalues greater than one and internal consistencies of high Cronbach α values.

## 4. Conclusions

This study evaluated the effects of processing (roasting and blanching) on the levels of phenolics, flavonoids and fatty acids in four Portuguese (Casanova, Molar, Pegarinhos and Refêgo) and two foreign (Ferragnès and Glorieta) almond cultivars, as well as on the antioxidant activities and sensorial characteristics of kernels. Antioxidant activities and levels of bioactive compounds were generally enhanced following roasting but reduced following blanching. The increased antioxidant activity upon roasting may at first seem unexpected, as heat could be envisaged to decrease (oxidize) phenolics. However, we interpret that roasting may either induce cell wall disruption allowing better antioxidant extraction, or may alternatively cause chemical alterations that result in heat-induced production of hyper-antiradical scavenging species. This might be linked to browning reaction s compounds, known to affect total phenolics quantification and antioxidant activities. Roasting and blanching reduced the ability of all cultivars to inhibit lipid oxidation. The fatty acid profiles of all cultivars were similar although raw kernels of Refêgo exhibited a high content of α-linolenic acid. Glorieta and Molar were characterized by a low and a high content of SFA respectively. Both roasting and blanching led to significant changes in the fatty acid profiles of almonds, similar for all cultivars with the exception of Refêgo, and the effect of blanching was stronger than that of roasting. Relative to health lipid indices, Pegarinhos and Molar had better responses to roasting and blanching than the other cultivars. Very few significant differences in cultivars and treatments relative to sensorial characteristics were found. The negative features bitter taste and bitter almond flavor were noticed for raw kernels of Molar and Pegarinhos. Roasting of Ferragnès led to kernels with a strong sweet almond flavor, while blanching positively affected the textural properties of Refêgo and Pegarinhos. The findings of this study shed light on the nutritive and eating qualities of raw and processed kernels from neglected Portuguese almond cultivars, and highlight the potential use of these cultivars in various food industries.

## Figures and Tables

**Table 1:** Total phenolic content, total flavonoid content, and antioxidant activities of raw and processed almond kernels (mean f.w., n = 3).

	Cultivar	Raw	Roasted	Blanched	*p* Value
Phenolics (mg GAE/g FW)	Casanova	0.09B a,b	0.49A b	0.04B b	0.001
	Ferragnès	0.06B b	0.58A b	0.05B a,b	0.001
	Glorieta	0.05B b	1.33A a,b	0.01B c	0.000
	Molar	0.09B a,b	1.16A a,b	0.02C b,c	0.007
	Pegarinhos	0.19B a B	0.88A a,b	0.08C a	0.003
	Refêgo	0.02B b	2.66A a	0.01C c	0.013
	*p* value	0.002	0.019	0.023	
Flavonoids (mg CE/g FW)	Casanova	0.76A b,c	1.23A a	0.09B	0.002
	Ferragnès	0.59A c	0.85A a,b	0.16B	0.033
	Glorieta	0.77A b,c	0.62A a,b	0.08B	0.020
	Molar	1.38A a,b	0.58A b	0.06C	0.000
	Pegarinhos	0.35A c	0.44A b	0.14B	0.000
	Refêgo	1.86A a	0.53A b	0.11B	0.001
	*p* value	0.000	0.014	0.578	
DPPH (µg Trolox/g)	Casanova	4.02B b	9.48A b	0.48C a,b	0.000
	Ferragnès	2.82B c	12.96A a	0.42C b	0.001
	Glorieta	1.54B d	4.86A b	0.70C a	0.016
	Molar	3.37A b,c	4.51A b	0.49B a,b	0.016
	Pegarinhos	6.42A a	7.60A b	0.64B a	0.004
	Refêgo	1.01A d	0.33B c	0.01C c	0.000
	p value	0.000	0.000	0.014	
ABTS (µg Trolox/g)	Casanova	8.81B a,b	13.96A a	0.47C b	0.000
	Ferragnès	5.07B c,d	14.23A a	0.56C a,b	0.000
	Glorieta	2.51B d,e	8.92A b	0.44C b	0.000
	Molar	7.27A b,c	8.05A b	0.52B a,b	0.000
	Pegarinhos	11.59A a	11.14A a,b	0.68B a	0.000
	Refêgo	1.56A e	2.64A c	0.41B b	0.009
	*p* value	0.000	0.000	0.004	
ß carotene bleaching assay (% inhibition)	Casanova	92.77A a,b	78.50B	71.41C	0.000
	Ferragnès	95.85A a,b	78.25B	70.79C	0.000
	Glorieta	96.22A a	80.65B	67.16C	0.000
	Molar	88.60A b,c	76.08B	69.75C	0.005
	Pegarinhos	84.89A 6c	71.33B	62.265C	0.000
	Refêgo	89.62A b,c	80.77A	67.91B	0.005
	*p* value	0.001	0.119	0.157	

Different small letters in front of mean within a column indicate significant differences among cultivars for the same treatment. Different capital letters in front of mean within a row indicate significant differences among treatments for the same cultivar (*p* < 0.05, ANOVA Tukey’s test).

**Table 2:** Contents of the main (most abundant and/or most affected) fatty acids in almond oil extracted from raw, roasted, and blanched kernels (%, mean, n = 3). Different small letters in front of mean within a row indicate significant differences among cultivars for the same treatment. Different capital letters in front of mean within a row indicate significant differences among treatments for the same cultivar (*p* < 0.05, ANOVA Tukey’s test). n.d.–not detected.

Cultivar		Casanova			Ferragnès			Glorieta			Molar			Pegarinhos			Refêgo	
	Raw	Roasted	Blanched	Raw	Roasted	Blanched	Raw	Roasted	Blanched	Raw	Roasted	Blanched	Raw	Roasted	Blanched	Raw	Roasted	Blanched
Palmitic	6.94A a	2.32B c	6.27A a	3.93A c	3.02AB c	2.71B c	2.92c	2.52c	2.96c	2.63B c	4.95A b	2.79B c	6.54A ab	2.14C c	3.57B bc	5.45B b	7.10A a	4.04C b
Stearic	0.132A a	n.d.	0.071B bc	n.d.	n.d.	0.36a	0.13a	n.d.	0.259ab	0.03B c	n.d.	0.33A a	0.11C b	0.17B a	0.23A ab	n.d.	0.09b	n.d.
Elaidic + Oleic	70.02A ab	34.05B d	68.26A a	61.51A c	48.28B b	45.97B c	77.03A a	40.86C c	54.11B b	65.08A bc	50.06B b	40.64C d	65.90A bc	26.87C e	42.67B cd	66.07B bc	82.19A a	52.17C b
Linoleic + Linolelaidic	13.54A ab	3.04B b	0.15C e	8.89A b	n.d.	1.05B a	10.34B ab	2.86C b	0.84A b	13.09A ab	0.36B c	0.54B cd	14.18A a	3.53B a	0.41C d	1.08A c	0.26C c	0.59B c
γ-Linolenic	0.17B	0.52A b	n.d.	0.104B	0.52B b	3.31A a	0.096C	0.75B a	2.31A b	0.08C	0.88B a	2.32A b	0.12C	0.48B b	1.66A c	n.d.	n.d.	0.943d
α-Linolenic	4.14C cd	33.89A b	18.89B b	4.99C bc	33.54A b	9.94B c	4.72B bc	32.73A b	6.18B cd	1.92B d	30.59A b	2.48B d	6.74C b	46.97A a	23.85B a	16.21A a	4.77B c	6.10B cd
cis-11,14-Eicosadienoic	n.d.	0.33b	n.d.	n.d.	n.d.	1.39a	n.d.	0.49B a	0.67A b	n.d.	n.d.	0.97b	n.d.	0.40B ab	0.67A b	n.d.	n.d.	0.69b
cis-8,11,14-Eicosatrienoic	n.d.	n.d.	0.10e	n.d.	n.d.	0.64c	n.d.	n.d.	1.42a	n.d.	0.27B b	0.83A b	n.d.	0.58A a	0.37B d	n.d.	n.d.	0.32d
cis-5,8,11,14,17-Eicosapentaenoic	n.d.	n.d.	0.10d	n.d.	n.d.	0.35c	n.d.	n.d.	0.79a	n.d.	0.29B b	0.62A ab	n.d.	0.48a	0.42bc	n.d.	n.d.	0.30cd
Erucic	1.97B ab	5.64A a	2.25B d	2.72B a	3.62B b	7.34A b	0.98B cd	5.73A a	5.73A c	0.51C d	3.44B b	9.35A a	1.41C bc	4.29B ab	5.81C c	2.47B a	2.59B b	6.32A bc
Nervonic	0.71B d	5.58A a	0.73B c	4.26A a	4.77A ab	3.31C b	1.06C c	4.18B bc	6.22A a	0.47C d	3.36B c	5.52A a	1.20C c	3.70B bc	5.18A a	2.31B b	0.69C d	5.29A a

**Table 3:** Contents of the main (most abundant and/or most affected) fatty acids in almond oil extracted from raw, roasted, and blanched kernels (%, mean, n = 3) Different small letters in front of mean within a row indicate significant differences among cultivars for the same treatment. Different capital letters in front of mean within a row indicate significant differences among treatments for the same cultivar (*p* < 0.05, ANOVA Tukey’s test). n.d.–not detected. SFA–saturated fatty acids; MUFA–monounsaturated fatty acids; PUFA–polyunsaturated fatty acids; AI–atherogenic index; TI–thrombogenic index; h/H–hypocholesterolemic/hypercholesterolemic index.

Cultivar		Casanova			Ferragnès			Glorieta			Molar			Pegarinhos			Refêgo	
	Raw	Roasted	Blanched	Raw	Roasted	Blanched	Raw	Roasted	Blanched	Raw	Roasted	Blanched	Raw	Roasted	Blanched	Raw	Roasted	Blanched
SFA	8.01B c	9.95A a	7.68B d	12.18A b	5.19C d	10.53B b	4.36C d	6.67B c	9.04A c	18.04A a	6.55C c	11.04B b	8.78A c	6.14B cd	8.74A cd	9.24B c	8.07C a	12.62A a
MUFA	70.73A ab	41.71B c	69.19A a	66.41A b	54.03B b	51.63B c	78.09A a	46.05C c	61.59B b	65.58A b	54.33B b	47.56C d	67.41A b	31.55C d	50.45B cd	68.58B b	82.88A a	61.32C b
PUFA	21.26B abc	48.34A b	23.14B c	20.88C abc	40.78A cd	37.84B a	17.55C bc	47.28A bc	29.36B b	16.37C c	39.12B d	41.39A a	23.81C a	62.1A a	40.80B a	22.17B ab	9.04C e	26.06A bc
PUFA/MUFA	0.30B ab	1.18A b	0.34B d	0.32B ab	0.76A c	0.733A b	0.225C b	1.03A bc	0.476B c	0.25C ab	0.72B c	0.872A a	0.35C a	1.98A a	0.81B ab	0.32B ab	0.11C d	0.425A cd
UFA/SFA	11.60A b	9.05B d	12.02A a	7.17C c	18.38A a	8.51B c	21.96A a	14.07B bc	10.08C b	4.57C d	14.38A bc	8.07B cd	10.39B b	15.28A ab	10.47B b	9.85B b	11.39A cd	6.929C d
AI	0.17A b	0.02C b	0.14A a	0.09A b	0.04B b	0.04B c	0.09A b	0.03B b	0.06A bc	0.73A a	0.06B b	0.04B c	0.14A b	0.01C b	0.05B c	0.13B b	0.43A a	0.08C b
TI	0.11A b	0.01C c	0.04B ab	0.06A c	0.02B c	0.03B b	0.05A c	0.02B c	0.04A b	0.16A a	0.03B b	0.03B b	0.09A b	0.01C c	0.03B b	0.05B c	0.09A a	0.05B a
hH	13.19B cd	34.22A ab	14.45B b	19.12b B	29.39b A	27.31A a	32.03A a	34.02A ab	27.29B a	9.08C d	17.83B c	24.82A ab	13.56C cd	39.91A a	22.38B ab	15.91B bc	12.70C c	18.46A ab
